# Cochrane corner: effectiveness of quarantine in reducing the spread of COVID-19

**DOI:** 10.11604/pamj.supp.2020.35.2.23051

**Published:** 2020-05-04

**Authors:** Jill Ryan, Akhona Victress Mazingisa, Charles Shey Wiysonge

**Affiliations:** 1 Cochrane South Africa, South African Medical Research Council, Cape Town, South Africa; 2 School of Public Health and Family Medicine, University of Cape Town, Cape Town, South Africa; 3 Department of Global Health, Stellenbosch University, Cape Town, South Africa

**Keywords:** COVID-19, pandemic, quarantine, Africa

## Abstract

**Introduction:**

there is no effective vaccine against coronavirus disease 2019 (COVID-19) at present, so non-pharmacological interventions like quarantine are advocated to control its spread. Quarantine refers to the restriction of the movement of asymptomatic healthy people who have had contact with cases of a communicable disease. We highlight a Cochrane rapid review, published in April 2020, on the effectiveness of quarantine in limiting the spread of COVID-19.

**Methods:**

the authors of the Cochrane rapid review searched multiple electronic databases for studies of any design, which assessed the effects of quarantine compared to no intervention. Eligible participants for the review included contacts of confirmed or suspected cases and people returning from countries with a declared outbreak of COVID-19, severe acute respiratory syndrome (SARS), or Middle East respiratory syndrome (MERS). The authors used the evidence from SARS and MERS studies to provide indirect evidence on COVID-19.

**Results:**

the authors included 29 observational and mathematical modelling studies and found that quarantine may lead to substantial reductions in new COVID-19 cases and deaths. The review also shows that combining school closures, travel bans and social distancing to quarantine may lead to larger reductions in cases and deaths.

**Conclusion:**

the review suggests that quarantine should be part of the COVID-19 combination prevention tool kit for Africa. Therefore, in addition to other public health measures, African countries should roll out COVID-19 testing to identify, isolate and treat infected people and quarantine their contacts.

## Introduction

Africa is facing a large-scale public health crisis, with coronavirus disease 2019 (COVID-19), the like of which we have never seen before. By 1st of May 2020, African countries had reported nearly 40,000 cases of COVID-19 [1]; with many countries in weeks of lockdown [2]. There is currently no effective vaccine against COVID-19 and many African countries have resorted to non-pharmacological public health interventions such as school closures, social distancing, isolation and quarantine to control the pandemic [3]. This commentary discusses a recent Cochrane rapid review by Nussbaumer-Streit and colleagues, on the effectiveness of quarantine in reducing COVID-19 spread and mortality [4]. Quarantine refers to the separation and restriction of movement for asymptomatic healthy people who have had contact with confirmed or suspected cases of a communicable disease [4].

## Methods

The authors included observational and mathematical modelling studies that assessed any type of quarantine alone or in combination with other interventions. Participants for this review included contacts of confirmed or suspected cases and people returning from countries with a declared outbreak of COVID-19, severe acute respiratory syndrome (SARS) or Middle East respiratory syndrome (MERS). The authors used the evidence from SARS and MERS studies to provide indirect evidence on COVID-19. They searched for studies in PubMed, Ovid MEDLINE, WHO Global Index Medicus, Embase, CINAHL and various Chinese databases up to 16 March 2020. The search strategy combined search terms for coronaviruses (such as coronavirus, COVID, MERS, SARS, etc) and terms related to quarantine (like quarantine, isolation, movement restriction, etc). The full search strategy is available in Appendix 1 and Appendix 2 of the Cochrane review (pages 39 to 41) [4]. The certainty of the evidence was evaluated using the Grading of Recommendations Assessment, Development and Evaluation (GRADE) method; which results in an assessment of the certainty of a body of evidence as high, moderate, low or very low [5].

## Results

**Figure 1:**
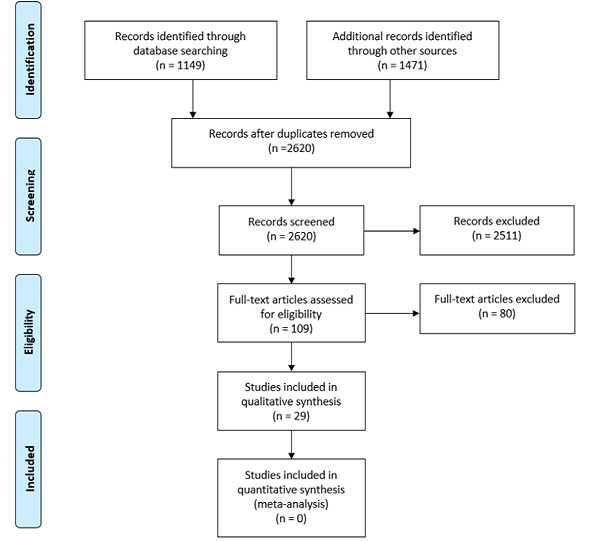
Flow diagram illustrating the search and selection of studies for the rapid review

The comprehensive search identified 2620 records, from which the authors included 29 eligible studies (23 published in English and 6 translated from Chinese). These included 25 modelling studies and four observational studies. Figure 1 summarises the search and selection of studies for the Cochrane review, in accordance with the Preferred Reporting Items for Systematic Reviews and Meta-Analyses (PRISMA) statement [6]. Compared to no intervention, the studies suggest that quarantine prevents 44-81% of new COVID-19 cases (4 studies) and 31-63% of COVID-19 related deaths (2 studies). The data also suggest that starting quarantine early may lead to larger reductions in new cases and deaths (2 studies) and greater cost savings (2 studies). Adding other interventions (like school closures, travel bans and social distancing) to quarantine may lead to larger reductions in new cases (4 studies) and deaths (2 studies).

## Discussion

The findings of this review suggest that quarantine, alone or in combination with the other public health interventions, has a vital role to play in containing COVID-19. The GRADE certainty of the evidence was downgraded to low for most outcomes because of study limitations and indirectness of the evidence (as some of the studies focused on SARS and not COVID-19). The low certainty of the evidence implies that the likelihood of further research finding the magnitude of the effectiveness of quarantine to be substantially different from the results of this review is high. In implementing a strict control measure such as quarantine, one needs to be cognisant of the context in which it is being implemented. There were no studies from Africa included in the review. Therefore, an impact evaluation is warranted when quarantine is implemented in African countries for the control of COVID-19.

Rigorous evaluations of quarantine for containment of COVID-19 in Africa should assess other important outcomes such as impacts on equity, cost implications and adverse effects. A recent rapid review found that quarantine may lead to post-traumatic stress symptoms, confusion and anger [7]. The information on COVID-19, its effects and interventions is still in development. With the disease spreading rapidly, the need for information is urgent. However, when reflecting upon the initial COVID-19 prevention measures as issued by World Health Organization such as improved hygiene and sanitation, this already proves challenging for some settings in Africa. Sub-Saharan Africa was one of two regions in the world which failed to meet the Millennium Development Goals for improved water infrastructure and sanitation facilities and the region continues to lag in decreasing deaths related to water and sanitation [8].

Seven out of ten Africans work in the informal sector and if COVID-19 control measures cause them further risks such as joblessness and food insecurity, there is a high likelihood of citizens defying containment measures [9]. Recent studies show that nation-wide testing during quarantine would be ideal, as it would mitigate long-term economic disruption by isolating infected persons and creating targeted quarantine for their contacts; thus, allowing the healthy workforce to continue economic activities [10]. If, however, it would be too costly or impractical to carry out wide-scale testing, then the benefits of quarantine would be sub-optimal [10].

## Conclusion

The Cochrane rapid review shows that quarantine may lead to large reductions in COVID-19 related morbidity and mortality. Therefore, African countries should roll out COVID-19 testing to identify, isolate and treat infected people and quarantine their contacts. However, quarantine needs to be considered in conjunction with the contextual needs of the implementing country. Its implementation needs to be accompanied by sensitive measures which can alleviate the brunt of poverty and other challenges currently experienced by citizens.

### What is known about this topic

- Quarantine has been used by various countries as a tactic for limiting the spread of COVID-19;
- No systematic review on the effects of quarantine had been published before the Cochrane rapid review.

### What this study adds

- Quarantine may lead to substantial decreases in infections and deaths from the COVID-19;
- The benefits of quarantine may be more pronounced if the intervention is started early.

## Competing interests

The authors declare no competing interests.
